# Public Health Perspectives on Aquaculture

**DOI:** 10.1007/s40572-014-0018-8

**Published:** 2014-07-15

**Authors:** Juan G. Gormaz, Jillian P. Fry, Marcia Erazo, David C. Love

**Affiliations:** 1 Molecular and Clinical Pharmacology Program, Institute of Biomedical Sciences, Faculty of Medicine, University of Chile, Independencia 1027, Región Metropolitana Santiago, Chile; 2 Johns Hopkins Center for a Livable Future, Bloomberg School of Public Health, Johns Hopkins University, 615 North Wolfe Street, Baltimore, MD 21205 USA; 3 Department of Environmental Health Sciences, Bloomberg School of Public Health, Johns Hopkins University, 615 North Wolfe Street, Baltimore, MD 21205 USA; 4 School of Public Health, Faculty of Medicine, University of Chile, Independencia 1027, Región Metropolitana Santiago, Chile; 5 Department of Nutrition, Faculty of Medicine, University of Chile, Independencia 1027, Región Metropolitana Santiago, Chile

**Keywords:** Aquaculture, Environment, Fish, Fish meal, Fish oil, Health, n-3 PUFA, Non-communicable diseases, Nutrition, Public health, Seafood

## Abstract

Nearly half of all seafood consumed globally comes from aquaculture, a method of food production that has expanded rapidly in recent years. Increasing seafood consumption has been proposed as part of a strategy to combat the current non-communicable disease (NCD) pandemic, but public health, environmental, social, and production challenges related to certain types of aquaculture production must be addressed. Resolving these complicated human health and ecologic trade-offs requires systems thinking and collaboration across many fields; the One Health concept is an integrative approach that brings veterinary and human health experts together to combat zoonotic disease. We propose applying and expanding the One Health approach to facilitate collaboration among stakeholders focused on increasing consumption of seafood and expanding aquaculture production, using methods that minimize risks to public health, animal health, and ecology. This expanded application of One Health may also have relevance to other complex systems with similar trade-offs.

## Introduction

Access to nutritious foods of animal origin, including aquatic animals, was crucial in the evolution of hominids and early human brain development [1•]. Aquatic animals contain essential nutrients, such as iodine and omega-3 long-chain polyunsaturated fatty acids (LCPUFAs), that are generally limited in other animal foods. While, historically, consumption of seafood has been important for humans, overfishing and other factors (e.g., population growth, pollution, ocean acidification) have greatly decreased wild fish stocks and damaged marine resources [2•]. In response to declining marine resources and an increasing demand for seafood, aquaculture has grown dramatically in the past four decades. In 2011, aquaculture accounted for nearly half of all seafood consumed by humans [3], and global aquaculture production continues to increase at a rate of 6 % per year [4]. Aquaculture production by country is depicted in Table 1.

**Table 1:** Global food fish aquaculture production by country in 2012 [121]

Country	Metric tons	Percentage of total
China, mainland	41,108,306	61.7 %
India	4,209,415	6.3 %
Viet Nam	3,085,500	4.6 %
Indonesia	3,067,660	4.6 %
Bangladesh	1,726,066	2.6 %
Norway	1,321,119	2.0%
Thailand	1,233,877	1.9 %
Chile	1,071,421	1.6 %
Egypt	1,017,738	1.5 %
Myanmar	885,169	1.3 %
Philippines	790,894	1.2 %
Brazil	707,461	1.1 %
Japan	633,047	1.0 %
Korea, RO	484,404	0.7 %
USA	420,024	0.6 %
Other countries	4,871,152	7.3 %
Total	66,633,253	100 %

**Fig. 1:**
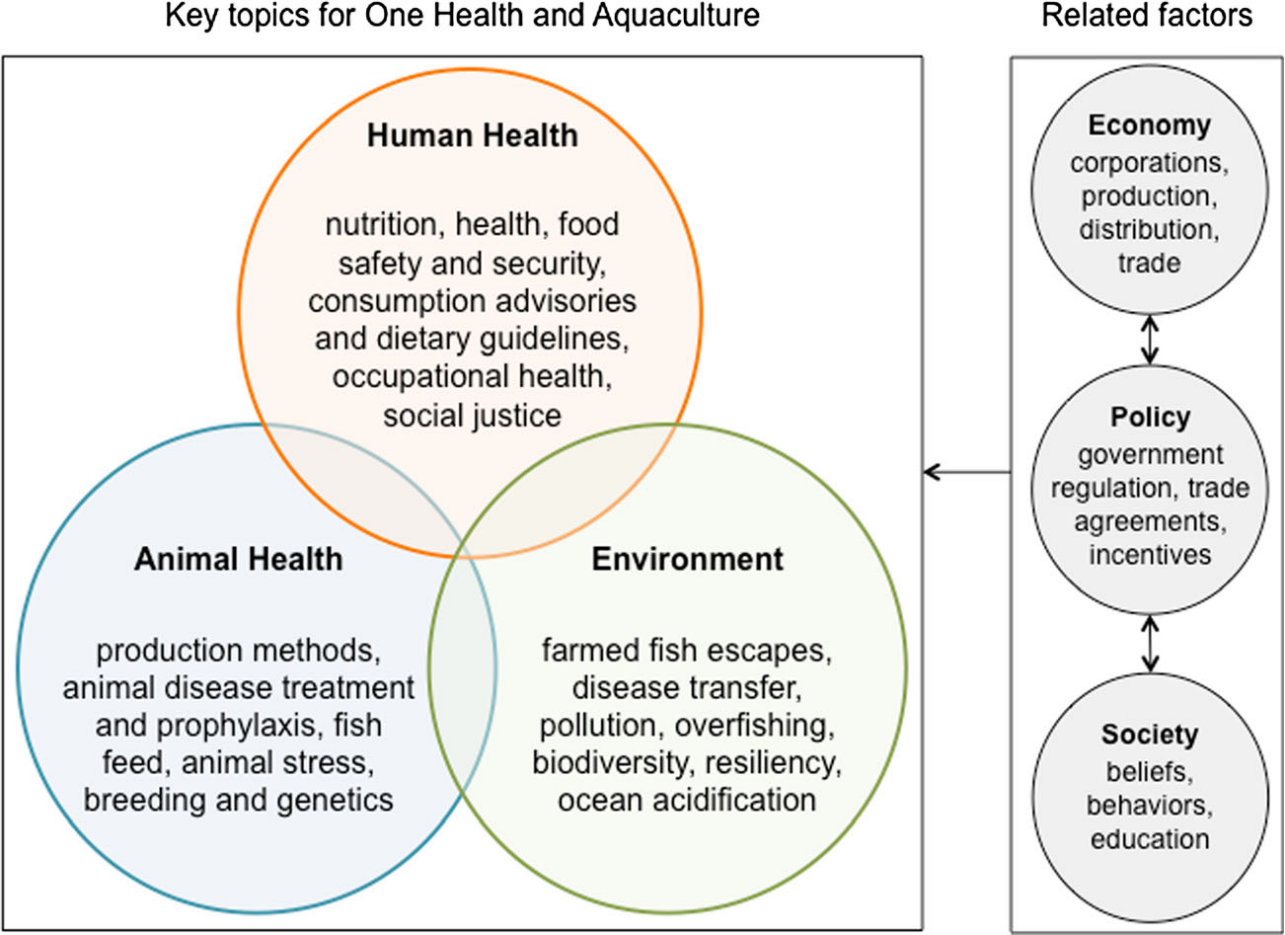
One Health and aquaculture

In many populations around the world, several factors have led to an overall shift in eating habits toward terrestrial livestock products and calorie-dense, nutrient-poor, highly processed foods [5, 6]. This shift, as well as other lifestyle and environmental factors, has contributed to a non-communicable disease (NCD) pandemic in many high-income, middle-income, and even some low-income countries [7–9]. In recent years, both the United Nations and the World Health Organization have recognized the global threat of NCDs and tried to strengthen national efforts to reduce their burden [10•, 11]. Seafood has been recognized as an important source of healthy dietary fats [12]. Many countries have developed a variety of dietary and physical activity recommendations for their citizens, many of which are related to dietary fat intake, such as eliminating intake of trans-fatty acids, reducing intake of saturated fats, and increasing consumption of healthy mono- and polyunsaturated fats, which can come from seafood [13–15]. The US government has issued dietary guidelines specifically promoting increased consumption of a variety of seafood in place of some meat and poultry [13]. Following seafood dietary guidelines may improve health for some, but increasing seafood consumption would require increased aquaculture production and wild-caught fish harvests. It may not be possible for wealthier nations to make progress on this recommendation without depleting global fisheries and further harming aquatic ecosystems, which could impact the food supplies of other nations [16]. Therefore, it is insufficient to simply increase seafood production without also taking equity and the protection of the public’s health and natural resources into account. To address these issues, we propose applying and expanding the One Health approach, which is an existing model for promoting synergy among the disciplines of human, animal, and environmental health sciences. Figure 1 describes several key topics for One Health and aquaculture, many of which are covered in this manuscript. Applying the One Health concept to aquaculture and the human health sciences could stimulate collaboration among scientists and other professionals who work on these issues.

The specific aims of this manuscript were to: (i) characterize the Western diet; (ii) examine the role of seafood in human nutrition and health; (iii) discuss challenges facing aquaculture and their implications for public health, the environment, and production; and (iv) describe how the One Health approach could be used as a convening tool for shaping decisions in regard to addressing the multiple and interrelated issues surrounding aquaculture, concerning nutrition, health, and the environment. In the context of a complex system, we provide examples of trade-offs between human health, environmental health, and seafood production, where the latest scientific evidence can help inform policy.

## Human Diet and Nutrition

### Western Diets and Health

For the majority of the populations in high- and middle-income countries, calories and macronutrients are easily accessible and relatively affordable, and physical activity is not essential for collecting food [6]. These conditions have contributed to the adoption of what is referred to as the Western diet—an eating pattern that is rich in empty calories and deficient in bioactive compounds, which commonly leads to weight gain and related health issues [17]. This dietary pattern is characterized as having a high percentage of unhealthy fats [18•] and has an unfavorable ratio of highly refined carbohydrates to complex carbohydrates, due to high starch and sugar content in many processed foods [19, 20]. In addition, the Western diet is the by-product of industrial agriculture and intensive animal production associated with elevated use of a variety of pesticides and chemotherapeutic agents. Industrial practices can unintentionally introduce chemical residues into the food supply [21, 22], and food engineering has incorporated a range of synthetic additives into foods [23].

These changes to our food system and diet, combined with largely sedentary lifestyles and other behavioral risk factors [11], have resulted in a pandemic of NCDs worldwide. In affected countries, NCDs are the leading causes of mortality, having replaced infectious diseases in what is known as the epidemiologic transition [24]. NCDs include a wide range of conditions with pathogenesis involving a strong oxidative–inflammatory component, which can result in both tissue death and cancer [25]. The most prevalent NCDs include obesity, metabolic syndrome, cardiovascular diseases, diabetes, and chronic respiratory diseases—including chronic obstructive pulmonary disease and cancer, which together are the leading causes of mortality globally (36 million deaths/year), with 80 % of NCD deaths occurring in low- and middle-income countries [26]. NCDs have become one of the greatest economic and societal challenges we face, making prevention of these diseases essential to improve the quality and duration of life [27].

Dietary prevention approach for NCDs mostly involve strategies for balancing caloric intake and expenditure to achieve and/or maintain a healthy weight and improving the nutritional quality of diets and food choices. The success of educational and behavioral interventions that promote the adoption of healthy nutrition habits is limited when environmental and social conditions that encourage these habits are not addressed [28]. This means that we ought to modify the structural issues related to the food system and promote healthy food production, distribution, marketing, and consumption [29]. Several researchers have suggested transitioning to a diet that replaces most terrestrial animal foods with aquatic foods [12, 18•, 30, 31] and emphasizing vegetables, fruits, and whole grains over highly refined sources of simple carbohydrates [19]. Given the realities of current production trends and declining fisheries, this aquatic food would increasingly come from aquaculture [3]. Other research indicates that a vegetarian, plant-based diet is another effective approach to reducing NCDs [32–34].

### The Role of Highly Processed Carbohydrates in Modern Western Diets

Consumption of highly processed carbohydrates has increased sharply over the last 50 years or so, because of advances in food technology and the ability of food ingredients such as fructose to induce overconsumption [20]. Increased intake of refined sugars and starch increases lipid storage and insulin resistance, a phenomenon closely linked to the development and progression of NCDs such as type 2 diabetes [18•]. Aquatic animal foods, on the other hand, lack carbohydrates and have a protein composition that favors a reduction in daily energy intake [35]. In addition, aquatic plants such as seaweed have a low energy contribution and are rich in micronutrients and bioactive compounds, like terrestrial leafy-green vegetables. Replacing a major proportion of the highly refined carbohydrates with whole grains and healthy aquatic plants and animals could alleviate some of the health risks associated with NCDs.

### The Role of Unhealthy Fats in Modern Western Diets

An increase in total fat consumption, especially of the so-called unhealthy fats such as cholesterol, trans- and saturated fatty acids (SFAs), combined with decreasing levels of healthy fats in Western diets, has been a major contributor to the current prevalence of NCDs [18•]. Historical (or ancestral) fat sources derived from aquatic food and animal nervous tissue have been replaced by other fats derived mainly from livestock, some crops, and their industrial by-products [1•, 30, 36]. These dietary changes have modified the proportions and dietary concentrations of all major groups of dietary fats, including fatty acids, sterols, and lipid-soluble vitamins [18•, 37, 38]. Of particular importance is the increase in the intake of animal sterols (mainly cholesterol), which may be associated with cardiovascular disease [37]. Animal sterols in the diet are mainly derived from foods high in terrestrial livestock fat, including full-fat dairy products.

Major dietary changes have also occurred in the levels and types of fatty acids, including SFAs, trans-fatty acids (TFAs), monounsaturated fatty acids (MUFAs), and polyunsaturated fatty acids (PUFAs), particularly LCPUFAs, which include omega-6 and omega-3 fatty acids. The incorporation of dietary TFAs from industrial sources (hydrogenation of unsaturated oils), as well as increases in the SFA to MUFA intake ratio, can also be associated with increased prevalence of cardiovascular diseases and other NCDs [18•]. The ratio between the two families of LCPUFAs (omega-6 and omega-3) has increased from historical ratios near 1:1 to 20:1 in Western populations, through both an increased intake of omega-6, mainly arachidonic acid (ARA; 20:4 n-6), and a decrease in the intake of omega-3, mainly docosahexaenoic acid (DHA) and its precursor eicosapentaenoic acid (EPA; 20:5 n-3) [30, 31]. This dramatic change in the omega-6 to omega-3 ratio has been associated with the development of NCDs [18•].

### Consumption of Aquatic Fats

Increasing the intake of omega-3 LCPUFAs (DHA and EPA) is emerging as a highly debated nutritional intervention to promote health in post-epidemiologic-transition countries [18•, 39, 40]. Currently, the main sources of these lipids are fatty or oily fish, shellfish, crustaceans, algae, and fortified foods. Supplements and food products fortified with LCPUFAs from fish oil or algae are increasingly available for people who consume little or no seafood. Levels of omega-3 LCPUFAs vary by fish species, with the highest amounts in salmon, anchovies, herring and shad, Atlantic or Pacific mackerel, bluefin or albacore tuna, and sardines (Table 2) [13]. The omega-3 LCPUFA present in aquatic animals comes from algae, which is consumed by small animals and then accumulates throughout the aquatic food chain [41].

**Table 2:** Characteristics of commonly consumed seafood in the USA

Species	Aquaculture component	US consumption (kg)^a^	Omega-3 content (EPA+DHA mg/4 oz)^b^
Shrimp	Yes	1.85	100
Canned light tuna	No	1.38	150–300
Salmon (Atlantic, chinook, coho, pink, sockeye)	Yes	0.975	700–2,400
Alaskan pollock	No	0.694	600
Tilapia	Yes	0.370	150
Catfish	Yes	0.461	100–250
Crab	Yes	0.284	200–550
Cod (Atlantic, Pacific)	No	0.245	200
Flatfish (flounder, plaice, sole)	No	0.183	350
Clams	Yes	0.215	200–300
Anchovies and herring	No	NA	2,300–2,400
Mackerel (Atlantic, Pacific)	No	NA	1,350–2,100

*DHA* docosahexaenoic acid, *EPA* eicosapentaenoic acid, *NA* not available

^a^Average of 2002–2008 [56]

^b^[13]

Fish oils and their principal bioactive components have been studied for nearly a century [18•]. The interest in these fatty acids related to the prevention of NCDs, however, started in the early 1970s, when a Danish biomedical research group reported that native Greenland Eskimos with high fat consumption and virtually no vegetable intake had a lower prevalence of cardiovascular disease and a complete absence of diabetes, compared with related urbanized Eskimos [42]. The native Eskimo diet, in spite of having moderate to slightly high levels of cholesterol and saturated fats, has high levels of EPA and DHA, and relatively low levels of ARA and dihomo-gamma-linolenic acid (DGLA) (20:3 n-6). As a result, plasma lipids in Eskimo people showed larger proportions of SFAs and omega-3, and smaller proportions of ARA and its omega-6 vegetal precursor linoleic acid (LA; 18:2 n-6) than those in their urbanized counterparts [43]. More recently, it has been shown that ARA and, indirectly, LA (major components of vegetable oils such as soybean oil, sunflower oil, and corn oil), may exert pro-inflammatory and thrombogenic effects, which could be antagonized by EPA and DHA. This reaffirms that an optimal dietary LCPUFA omega-6 to omega-3 ratio may have been the major cause of the low prevalence of NCDs in native Greenland Eskimos [18•].

### The Marine Oils Paradox

Despite the wealth of basic, clinical, and epidemiologic evidence indicating that diets rich in fish oil and related supplementation can prevent coronary heart disease (CHD) [12] and treat some cardiovascular risk factors such as hypertriglyceridemia and inflammation [44], there are several studies that have reported no effects [45]. In addition, the potential benefits of omega-3 LCPUFA consumption in the prevention of metabolic-related NCDs such as type 2 diabetes, rather than CHD and fatty liver disease [46], remain to be demonstrated with more epidemiologic studies [47].

These inconsistences could be attributable to several factors. First, the effects of pollutants present in the dietary fatty fish and fish oil supplements consumed by the study populations may have masked omega-3 LCPUFAs’ beneficial effects. In one study, the authors found that mercury masked the inverse association between DHA levels and the risk of myocardial infarction [48]. Second, the use of supplement formulations with a non-optimal EPA to DHA ratio could have led to inconsistent findings [49]. Third, many of the current studies may have had methodological weaknesses. For example, the DART-2 study [50] was not blinded and used a supplement containing EPA-rich oil derived from fish, without reporting anything about DHA levels. Other studies only considered omega-3 intake without considering dietary omega-6 levels and other dietary factors [51]. These methodological weaknesses maybe explain why Dyerberg and Bang found a strong association between dietary omega-3 LCPUFAs and a low prevalence of CHD in native Greenland Eskimos [42], while other studies failed to show a protective effect of these lipids on those diseases. Because of these contradictions, researchers should continue to investigate the differences between the effects of omega-3 LCPUFAs in acute or chronic diseases, as well as the optimal timing and doses of omega-3 LCPUFAs [52]. To address methodological weaknesses, new and more robust studies are needed to establish a solid association between omega-3 LCPUFAs and NCD-associated metabolic disorders, beyond CHD. In addition, there is a growing awareness of the need to balance the use of fish oils with the environmental impacts of creating fish oil, due to overfishing and other factors [40], which is an example of scientists using an expanded One Health-type approach in considering dietary recommendations.

### Non-fatty Seafood Consumption, More Than Omega-3 LCPUFAs

After health benefits of fish consumption were discovered in populations that consume large quantities of fatty fish and marine mammals, seafood nutrition research focused on omega-3 LCPUFAs because these fatty acids are a major component in some seafood and Western diets are low in omega-3 LCPUFAs. In the USA, however, the most commonly consumed aquatic species are shrimp, canned tuna, salmon, pollock, tilapia, and catfish. Most of these products contain low levels of omega-3 LCPUFAs, with the exception of salmon (Table 2). Consumer preference in the USA for these products suggests that factors other than omega-3 LCPUFAs affect seafood purchasing. Many nutritional guidelines focus on “fatty fish” when recommending an increase in seafood consumption, because of the large variation in levels of omega-3 LCPUFAs in seafood products.

The low levels of omega-3 LCPUFAs in lean seafood, however, do not mean that these products are unhealthy. Epidemiologic evidence has shown that health benefits of CHD attributed to seafood consumption were associated with a modest intake of seafood products [53]. Lean marine food is considered to be an excellent source of some key nutrients, having higher omega-3 LCPUFA levels and lower levels of total unhealthy fats than livestock products (i.e., meat, sausages, and cold cuts); therefore, replacing the former with the latter can result in significant beneficial effects on cardiovascular health [54]. To make a significant impact on health at a population level, the current average consumption of seafood (6.8 kg/person/year) in the USA would have to be significantly higher and must be accompanied by a reduction in the intake of unhealthy foods and increased consumption of nutrient-rich vegetables [19, 55, 56].

## Public Health, Environmental, and Production Challenges Facing Aquaculture

### Public Health Challenges: Contaminants in Seafood

Contaminants exist in wild and farmed seafood. The nature of contamination depends upon the species, geographic region, animal age and diet, and production practices. Among farmed seafood, the main contaminants of concern are methylmercury, persistent organic pollutants (POPs), and production drugs (production drugs are discussed in section 3.1.2). Farmed fish acquire POPs and mercury from consuming fish meal and oil, which are feed products made from small, wild pelagic fish such as herring and sardines. POPs and mercury in the ocean come from atmospheric deposition of emissions, mainly from the combustion of fossil fuels for mercury and agricultural pesticide application for POPs [57, 58]. POPs and mercury in coastal waters come from discharge/runoff from industrial sources and from contaminated land and sediments [57, 58]. Farmed salmon tends to have lower levels of POPs and mercury when fish-based feed is sourced from regions of the world where small pelagic fish have fewer contaminants, such as South America, compared with small pelagic fish from Northern Europe, which have higher levels of contaminants [59, 60]. Some fish processors are implementing activated carbon filters to remove POPs from fish meal and oil as a means of reducing contaminants in farmed salmon [61]. Another approach to reducing POPs in farmed fish is to reduce the use of fish meal and oil in aquaculture.

Among wild-caught fish, methylmercury, heavy metals, and POPs are the most concerning contaminants with regard to public health. These contaminants are primarily found in apex predatory fish such as shark or swordfish, and in bottom feeders such as tilefish. These fish acquire contaminants as a result of bio-magnification up the food web, and from decades of intensive industrial development in the northern hemisphere. Consuming these fish is not advised by national governments [13], and epidemiologic studies have quantified the risks from consuming these fish, particularly among vulnerable groups such as pregnant women, in populations that consume large amounts of seafood [62]. Levels of mercury, other heavy metals, and POPs in wild-caught fish are generally lower in the southern hemisphere, but are increasing [63].

Scientists have attempted to assess the trade-offs between the health risks associated with heavy metals and POPs in seafood and the benefits of omega-3 LCPUFAs [12]. One study reported that high levels of mercury can mask the beneficial cardiovascular effects of DHA [48]. Another review article concluded that the cardiovascular benefits of regular intake of farmed salmon with high levels of omega-3 LCPUFAs outweigh the theoretical risks associated with chronic exposure to moderate levels of polychlorinated biphenyls (PCBs) [12]. The findings of Roth and Harris [12] align with a previous systematic review showing that for adults, the benefits of fish consumption outweigh the risks [64]. In women of childbearing age, the benefits of seafood intake also outweigh the risks, if some species high in mercury and POPs are not consumed [64]. In a recent systematic review, positive associations were found between prenatal or postnatal seafood intake and child neurodevelopment in most studies; however, the counterbalancing effects of pollutants (POPs and methylmercury) and health benefits can make it challenging to observe health benefits [65]. Risk communication about seafood consumption is evolving as the science on health effects evolves. Unfortunately, both consumers and the media have been confused by public health messaging about the benefits and risks of seafood, which may suppress consumption among groups such as pregnant women, who could benefit from eating non-contaminated seafood high in omega-3 LCPUFAs [66, 67•, 68].

### Public Health Challenges: Aquaculture and Production Drugs

A variety of chemicals (algaecides, antibiotics, disinfectants, herbicides, pesticides, and probiotics) are used in aquaculture to treat and prevent diseases, in an attempt to achieve maximum production [69, 70]. Contamination of farmed seafood products and the environment due to the use of these chemicals can cause risks to human health, potentially increasing the prevalence of some NCDs such as cancer [71]. Antibiotics used in medicated feed can diffuse into the water column, spreading to sediments and wild fauna [72–74]. In laboratory trials, some antibiotics have remained stable and retained antimicrobial activity for months in marine sediments [75], and the insecticide diflubenzuron has remained stable for at least 7 months in sediment [76]. Research has documented an increase in antibiotic-resistant bacteria associated with antibiotic use in aquaculture [77–79]. The USA, European Union, Chile, and many other countries inspect only a fraction of commercial products for environmental chemicals, microorganisms, pesticides, and veterinary drugs to safeguard the food supply [80]. Given the human health risks associated with the use of chemicals and veterinary drugs in aquaculture, this problem fits well into a One Health framework. To address this issue, these drugs should be used minimally, and veterinary drugs should be used under the supervision of a veterinarian and regulated by the appropriate government agency. In addition, investment in research aimed at understanding the underlying causes and epidemiology of animal diseases is critical for ultimately reducing the use of production drugs.

### Public Health Challenges: Social Impacts and Inequity

Industrial-scale aquaculture products are largely exported to other countries or sold to middle- or high-income individuals in the country of production, resulting in situations in which people living near aquaculture production are impoverished and food insecure [81]. This is especially true in the case of populations that previously used the land/water for subsistence fishing, as is true for many sites now used for industrial shrimp farming in Southeast Asia [82]. Environmental impacts from aquaculture can affect the well-being of coastal communities by changing residents’ sense of place, decreasing community involvement, and compromising mental health [83]. In addition, aquaculture’s use of fish meal and oil competes with the availability of fish for human consumption [84]. The dependency on fish for food is higher on islands and in coastal communities, especially in low-income countries, where fish and other seafood represent a high proportion of dietary animal protein [85]. For these populations, small fish are the main source of micronutrients that help to combat dietary deficiencies [86•, 87]. Alternatively, aquaculture can help people living in poverty by providing employment and sustaining local economies; this may be especially true for women, who constitute much of the post-harvest aquaculture workforce [88]. Therefore, it is essential to examine aquaculture operations on the basis of their impact on living conditions and how/if they meet the needs of consumers at various income levels, both proximal to the production site and for export markets.

### Environmental Challenges: Aquaculture and Environmental Health

Maintaining a high degree of environmental quality is critical to aquaculture, because of multiple ecologic feedback loops linking human health and seafood production. Aquaculture may affect human health and nutrition by reducing wild fish populations (because of its use in aquaculture feed) or by causing environmental impacts and spreading fish diseases that reduce future aquaculture or fisheries production. Scarcity of wild fish and/or aquaculture products would not only impact food security but could also increase consumption of foods that promote the development of NCDs.

A significant challenge facing aquaculture is increasing seafood production to the levels needed to positively impact diets at a population level without degrading aquatic ecosystems. In addition to overfishing concerns related to feed ingredients, there are impacts on the local environment at many aquaculture sites, associated with the chemicals used on farms, effluent discharges and water quality, disease transmission between farmed and wild species, concentration of fish waste, and fish escapes [4, 89–92].

Production systems that interface directly with the environment and rely on ecosystem services, such as clean water and fish waste decomposition, have a greater likelihood of negatively impacting their surroundings, compared with recirculating systems that are sited on land. This is perhaps why policies and siting of new offshore aquaculture operations are particularly contentious [93, 94]. Therefore, a better understanding is needed regarding the trade-offs between different aquaculture production methods such as offshore and recirculating systems [95].

### Environmental Challenges: Aquaculture and Animal Health

Each year, aquaculture producers lose large amounts of farm-raised seafood because of infectious disease outbreaks, which cost billions of dollars, impact international trade, and generate negative publicity for the aquaculture industry [96, 97]. Over the past 30 years in Southeast Asia and South America, over a dozen emerging viral diseases have spread throughout shrimp farms, some causing very high mortality rates [98]. Over the past few years, infectious salmon anemia (ISA) [99], a viral disease, has impacted Chile by causing significant reductions in salmon production and exports [100]. Although most aquaculture diseases do not directly impact human health because the pathogens do not infect humans, the chemicals and drugs used to prevent or treat them can impact the environment and public health.

A variety of factors contribute to emerging diseases in aquaculture, including (i) globalization and international trade; (ii) consolidation and intensification of hatcheries and production; (iii) introduction of hatchery-raised species to new environments; (iv) interactions between wild and farmed animals; (v) biosecurity; and (vi) climate change [97, 101]. Specific risk factors have been developed for particular species, regions, production methods, and diseases. In Chilean salmon culture, risk factors for infectious salmon anemia were related to insufficient surveillance and diagnostic efforts, poor management practices, close proximity of farms, high prevalence of sea lice (*Caligus rogercresseyi*), insufficient disease prevention and contingency plans, poor stock quality control, and insufficient transportation practices [102]. Additional issues include potentially suboptimal feed formulation and a scarcity of local research and development in fish nutrition [103]. In Southeast Asian shrimp production, risk factors for white spot syndrome virus include high stocking densities, use of wild stocks raised in ponds, use of alternative and live feeds, poor water quality, and animal stress [98].

Given the complex set of interactions that facilitate the spread of disease, multi-level interventions are necessary. Farm-level disease interventions, such as timely diagnosis and treatment, could address the host–pathogen relationship, while environmental stewardship and improved farm management may address environment–pathogen and environment–host issues. To address disease transmission between farms, regional and national policies, surveillance, reporting, training, and emergency response capabilities are also needed [97].

### Production Challenges: Reducing Aquaculture’s Dependence on Fisheries

In 2000, Sena S. De Silva, an aquaculture expert at Deakin University in Australia, predicted a paradigm shift in aquaculture, from “an increased production at almost any cost, to a sustainable increase in production with minimal environmental perturbations” [104]. When putting De Silva’s words into practice, a major challenge for aquaculture companies is to acquire aquaculture feed for a growing industry without depleting wild fish stocks. Aquaculture feeds are made with small pelagic forage fish, in part to produce farmed fish with levels of omega-3 LCPUFAs that are equivalent to those of their wild counterparts [91]. These forage fish come from a global supply that is expected to remain static or decrease over time, while increasing in price [91, 105].

In the past few decades, fish meal and oil from forage fish have been steadily reduced in aquaculture feed and replaced with plant-based ingredients such as soybean meal, which can reduce pressure to harvest small pelagic forage fish. However, some carnivorous fish cannot easily metabolize high levels of plant protein [106]. In addition, these plant-based ingredients could substantially reduce omega-3 LCPUFA levels in farmed seafood and change the overall fat composition of the product, reducing their nutritional benefits [107, 108]. These plant-based diets could also affect aquaculture productivity by undermining the health and immune resistance of the fish [109, 110]. Dramatically increasing demand for ingredients in plant-based feeds may have collateral impacts on water and pesticide use, eutrophication, and climate change, as a result of increasing production of soy and other crops [111].

Possible alternatives to fish meal and oil from wild-caught sources are by-products from seafood processing (i.e., heads, bones, tails, etc.) and non-edible by-catch that would otherwise be discarded. Single-celled microalgae have been tested in feeding trials as a fish oil replacement, with some success, although adoption would require large-scale production at competitive prices [112, 113]. Cobia, a carnivorous species in the wild, has been successfully raised on a vegetarian feed supplemented with soybean oil and small marine organisms (microheterotrophs) similar to algae [114•]. As algae production is expected to increase, there may be competition for using algae as either a feed or a biofuel, similar to corn and other crops [115]. Livestock by-products (rendered animal carcasses) are also being evaluated as a protein replacement in fish and shrimp production; certain animal proteins (pork, poultry) have recently been approved for use in the European Commission as aquaculture feed [116] and have always been allowed in the USA. There are public health concerns surrounding the use of poultry feather meal, a rendered by-product, as a feed ingredient for fish, because of the presence of veterinary drug residues [22]. Additional research is needed to address a variety of public health concerns around aquaculture feed ingredients such as soy and animal by-products.

## Aquaculture Development Moving Forward: Adopting and Expanding the One Health Concept

As with any type of agriculture, aquaculture interacts with human social systems and ecological systems, and this can lead to conflicts over natural resource use and environmental impacts [117]. There is a wide range of stakeholders involved with aquaculture, including industry groups, scientists, government agencies, nongovernmental organizations, and consumers, who each bring their own perspectives to the topic. Aquaculture producers are generally driven by profitability and long-term economic sustainability, which may include minimizing environmental impacts. Some aquaculture companies have taken steps and publicized plans to address a variety of environmental concerns [118], although governments, nongovernmental organizations, and consumers should hold them accountable regarding proposed plans and timelines. Human health professionals have historically been minimally involved with issues related to aquaculture, perhaps because of single-discipline focused research. Communicating the connections between aquaculture and public health could increase their involvement. We propose involving the medical and public health communities with aquaculture by applying and expanding the One Health concept. As stated earlier, this is a systems approach for linking human, animal, and environmental health issues relevant to a single topic [119]. One Health has historically focused on infectious diseases that pass between animals and humans, although recently others have suggested that food safety and food production are priority areas where the One Health concept could be applied [120]. A variety of disciplines, including research, clinical medical and veterinary practice, and policy, can be applied to aquaculture and seafood via One Health (Fig. 1). Animal health and environmental impacts are particularly important because they remain major constraints to production of aquaculture that could affect people’s access to healthy food [97, 117].

In this manuscript, we have highlighted several cross-cutting topics that are relevant to aquaculture and One Health, including: (i) balancing the issues of increased fish consumption for health reasons (such as reducing NCDs), overfishing of wild-caught seafood, and increasing aquaculture production with minimal environmental impacts; (ii) identifying and communicating the human health risks from contaminants in seafood, and strategies to mitigate those risks; (iii) developing and promoting production methods that reduce or eliminate the need for antibiotics, pesticides, and other chemicals, which can have wide-ranging impacts on human, fish, and environmental health; and (iv) finding ecologically sustainable and safe animal feeds with acceptable levels of healthy nutrients and bioactive compounds (i.e., marine omega-3 fatty acids) without contributing to overfishing or compromising human food security for low-resource coastal communities. These are just a few of the most pressing issues that could benefit from collaboration with experts in the medical, public health, and veterinary communities.

## Conclusion

The current epidemiologic profile of the global population shows increasing rates of NCDs. The health benefits associated with regular consumption of moderate amounts of seafood in place of meats, and increased consumption of vegetables and fruits, could help reduce rates of NCDs. Recommendations for increasing seafood consumption must be balanced with risks of further damage to fisheries, and other risks to public health and the environment from some forms of aquaculture. Therefore, examining the interactions between aquaculture, fisheries, human diet and health, and ecological health can assist in setting priorities for enhancing human nutrition and the ecological sustainability of aquaculture. We propose applying and expanding the One Health model to engage experts in a variety of fields to collaborate on developing methods and policies that will allow aquaculture to operate sustainably and contribute to human diets that promote health.

## References

[1.•] Crawford MA, Broadhurst CL (2012). The role of docosahexaenoic and the marine food web as determinants of evolution and hominid brain development: the challenge for human sustainability. Nutr Health.

[2.•] Froese R, Zeller D, Kleisner K, Pauly D (2012). What catch data can tell us about the status of global fisheries. Mar Biol.

[3] Food and Agriculture Organization of the United Nations (2012). The state of world fisheries and aquaculture.

[4] Bostock J, McAndrew B, Richards R (2010). Aquaculture: global status and trends. Philos Trans R Soc Lond Ser B Biol Sci.

[5] Feskens EJ, Sluik D, van Woudenbergh GJ (2013). Meat consumption, diabetes, and its complications. Curr Diabetes Rep.

[6] Stuckler D, McKee M, Ebrahim S, Basu S (2012). Manufacturing epidemics: the role of global producers in increased consumption of unhealthy commodities including processed foods, alcohol, and tobacco. PLoS Med.

[7] Gilbert PA, Khokhar S (2008). Changing dietary habits of ethnic groups in Europe and implications for health. Nutr Rev.

[8] Pan A, Sun Q, Bernstein AM (2012). Red meat consumption and mortality: results from 2 prospective cohort studies. Arch Intern Med.

[9] Alwan A. Global status report on noncommunicable diseases 2010. World Health Organization; 2011: 176.

[10.•] United Nations. Political declaration of the High-Level Meeting of the General Assembly on the Prevention and Control of Non-communicable Diseases*.* Edited by General Assembly. Vol. 66; 2012. *In this resolution, the UN recognizes that NCDs are a global threat, agreeing to prioritize the prevention and control of this burden.*

[11] World Health Organization. Development of an updated action plan for the global strategy for the prevention and control of noncommunicable diseases covering the period 2013 to 2020. 2012.

[12] Roth EM, Harris WS (2010). Fish oil for primary and secondary prevention of coronary heart disease. Curr Atheroscler Rep.

[13] Dietary guidelines for Americans. Washington DC: US Department of Agriculture and US Department of Health and Human Services; 2010.

[14] Olivares SC, Zacarias IH. Guias alimentarias para la población Chilena. Edited by Health Mo. Chile; 2013.

[15] National Nutrition Council. Nutrition recommendations 2014. Edited by Ministry of Agriculture and Forestry; 2014.

[16] Brunner EJ, Jones PJS, Friel S, Bartley M (2009). Fish, human health and marine ecosystem health: policies in collision. Int J Epidemiol.

[17] Pijl H (2011). Obesity: evolution of a symptom of affluence. Neth J Med.

[18.•] Gormaz JG, Erazo M, Ferreira JE, Muniz N (2012). Dietary fat and its impact on health: analysis of basic, clinical and epidemiological evidence. Low and high-fat diets: myths vs reality.

[19] Aller EE, Abete I, Astrup A, Martinez JA, van Baak MA (2011). Starches, sugars and obesity. Nutrients.

[20] Johnson RJ, Segal MS, Sautin Y (2007). Potential role of sugar (fructose) in the epidemic of hypertension, obesity and the metabolic syndrome, diabetes, kidney disease, and cardiovascular disease. Am J Clin Nutr.

[21] Neff RA, Hartle JC, Laestadius LI, Dolan K, Rosenthal AC, Nachman KE (2012). A comparative study of allowable pesticide residue levels on produce in the United States. Glob Health.

[22] Love DC, Halden RU, Davis MF, Nachman KE (2012). Feather meal: a previously unrecognized route for reentry into the food supply of multiple pharmaceuticals and personal care products (PPCPs). Environ Sci Technol.

[23] Seiber JN, Kleinschmidt L (2012). From detrimental to beneficial constituents in foods: tracking the publication trends in JAFC. J Agric Food Chem.

[24] Hanson M, Godfrey KM, Lillycrop KA, Burdge GC, Gluckman PD (2011). Developmental plasticity and developmental origins of non-communicable disease: theoretical considerations and epigenetic mechanisms. Prog Biophys Mol Biol.

[25] Rodrigo R (2009). Oxidative stress and antioxidants: their role in human disease.

[26] World Health Organization. Noncommunicable diseases country profiles. 2011.

[27] Probst-Hensch N, Tanner M, Kessler C, Burri C, Künzli N (2011). Prevention—a cost-effective way to fight the non-communicable disease epidemic: an academic perspective of the United Nations High-Level NCD Meeting. Swiss Med Wkly.

[28] Cismaru M (2008). Counteracting obesity: developing a policy framework to guide action. Int J Publ Health.

[29] Fan S, Pandya-Lorch R (2012). Reshaping agriculture for nutrition and health.

[30] Simopoulos AP (2011). Evolutionary aspects of diet: the omega-6/omega-3 ratio and the brain. Mol Neurobiol.

[31] Molendi-Coste O, Legry V, Leclercq IA (2011). Why and how meet n-3 PUFA dietary recommendations?. Gastroenterol Res Pract.

[32] Sabaté J (2003). The contribution of vegetarian diets to health and disease: a paradigm shift?. Am J Clin Nutr.

[33] Huang T, Yang B, Zheng J, Li G, Wahlqvist ML, Li D (2012). Cardiovascular disease mortality and cancer incidence in vegetarians: a meta-analysis and systematic review. Ann Nutr Metab.

[34] Craig WJ (2010). Nutrition concerns and health effects of vegetarian diets. Nutr Clin Pract: Off Publ Am Soc Parenter Enter Nutr.

[35] Borzoei S, Neovius M, Barkeling B, Teixeira-Pinto A, Rossner S (2006). A comparison of effects of fish and beef protein on satiety in normal weight men. Eur J Clin Nutr.

[36] Ferraro JV, Plummer TW, Pobiner BL (2013). Earliest archaeological evidence of persistent hominin carnivory. PLoS One.

[37] Rifkind BM (1984). Lipid Research Clinics Coronary Primary Prevention Trial: results and implications. Am J Cardiol.

[38] Niki E, Traber MG (2012). A history of vitamin E. Ann Nutr Metab.

[39] von Schacky C (2014). Omega-3 index and cardiovascular health. Nutrients.

[40] Greene J, Ashburn SM, Razzouk L, Smith DA (2013). Fish oils, coronary heart disease, and the environment. Am J Public Health.

[41] Gladyshev MI, Sushchik NN, Makhutova ON (2013). Production of EPA and DHA in aquatic ecosystems and their transfer to the land. Prostaglandins Other Lipid Mediat.

[42] Dyerberg J, Bang HO (1979). Haemostatic function and platelet polyunsaturated fatty acids in Eskimos. Lancet.

[43] Dyerberg J, Bang HO, Hjorne N (1975). Fatty acid composition of the plasma lipids in Greenland Eskimos. Am J Clin Nutr.

[44] Lopez-Huertas E (2012). The effect of EPA and DHA on metabolic syndrome patients: a systematic review of randomised controlled trials. Br J Nutr.

[45] Rizos EC, Ntzani EE, Bika E, Kostapanos MS, Elisaf MS (2012). Association between omega-3 fatty acid supplementation and risk of major cardiovascular disease events: a systematic review and meta-analysis. JAMA.

[46] Parker HM, Johnson NA, Burdon CA, Cohn JS, O'Connor HT, George J (2012). Omega-3 supplementation and non-alcoholic fatty liver disease: a systematic review and meta-analysis. J Hepatol.

[47] Wu JH, Micha R, Imamura F (2012). Omega-3 fatty acids and incident type 2 diabetes: a systematic review and meta-analysis. Br J Nutr.

[48] Guallar E, Sanz-Gallardo MI, van't Veer P (2002). Mercury, fish oils, and the risk of myocardial infarction. N Engl J Med.

[49] Rodrigo R, Korantzopoulos P, Cereceda M (2013). A randomized controlled trial to prevent postoperative atrial fibrillation by antioxidant reinforcement. J Am Coll Cardiol.

[50] Burr ML, Ashfield-Watt PA, Dunstan FD (2003). Lack of benefit of dietary advice to men with angina: results of a controlled trial. Eur J Clin Nutr.

[51] Wood KE, Lau A, Mantzioris E, Gibson RA, Ramsden CE, Muhlhausler BS (2014). A low omega-6 polyunsaturated fatty acid (n-6 PUFA) diet increases omega-3 (n-3) long chain PUFA status in plasma phospholipids in humans. Prostaglandins Leukot Essent Fatty Acids.

[52] Kumar S, Sutherland F, Lee JM (2013). Effects of high dose intravenous fish oil on human atrial electrophysiology: implications for possible anti- and pro-arrhythmic mechanisms in atrial fibrillation. Int J Cardiol.

[53] Virtanen JK, Mozaffarian D, Chiuve SE, Rimm EB (2008). Fish consumption and risk of major chronic disease in men. Am J Clin Nutr.

[54] He K (2009). Fish, long-chain omega-3 polyunsaturated fatty acids and prevention of cardiovascular disease—eat fish or take fish oil supplement?. Prog Cardiovasc Dis.

[55] Raatz SK, Silverstein JT, Jahns L, Picklo MJ (2013). Issues of fish consumption for cardiovascular disease risk reduction. Nutrients.

[56] Top 10 consumed seafoods [http://www.aboutseafood.com/about/about-seafood/top-10-consumed-seafoods]

[57] Lohmann R, Breivik K, Dachs J, Muir D (2007). Global fate of POPs: current and future research directions. Environ Pollut.

[58] Chen CY, Driscoll CT, Lambert KF, Toxic Metals Superfund Research Program (2012). Sources to seafood: mercury pollution in the marine environment.

[59] Ikonomou MG, Higgs DA, Gibbs M (2007). Flesh quality of market-size farmed and wild British Columbia salmon. Environ Sci Technol.

[60] Hites RA, Foran JA, Carpenter DO, Hamilton MC, Knuth BA, Schwager SJ (2004). Global assessment of organic contaminants in farmed salmon. Science.

[61] Hardy RW, Lee C-S (2010). Aquaculture feed and seafood quality. Bull Fish Res Agen.

[62] Dallaire R, Dewailly E, Ayotte P (2013). Exposure to organochlorines and mercury through fish and marine mammal consumption: associations with growth and duration of gestation among Inuit newborns. Environ Int.

[63] Hermanns YM, Biester H (2013). Anthropogenic mercury signals in lake sediments from southernmost Patagonia, Chile. Sci Total Environ.

[64] Mozaffarian D, Rimm EB (2006). Fish intake, contaminants, and human health: evaluating the risks and the benefits. JAMA: J Am Med Assoc.

[65] Avella-Garcia C, Julvez J (2014). Seafood intake and neurodevelopment: a systematic review. Curr Environ Health Rpt.

[66] Greiner A, Clegg Smith K, Guallar E (2010). Something fishy? News media presentation of complex health issues related to fish consumption guidelines. Public Health Nutr.

[67.•] Teisl MF, Fromberg E, Smith AE, Boyle KJ, Engelberth HM (2011). Awake at the switch: improving fish consumption advisories for at-risk women. Sci Total Environ.

[68] Oken E, Kleinman KP, Berland WE, Sitnn SR, Rich-Edwards JW, Gillman MW (2003). Decline in fish consumption among pregnant women after a national mercury advisory. Obstet Gynecol.

[69] Graslund S, Bengtsson B (2001). Chemicals and biological products used in South-East Asian shrimp farming, and their potential impact on the environment—a review. Sci Total Environ.

[70] Burridge L, Weis JS, Cabello F, Pizarro J, Bostick K (2010). Chemical use in salmon aquaculture: a review of current practices and possible environmental effects. Aquaculture.

[71] Sankpal UT, Pius H, Khan M (2012). Environmental factors in causing human cancers: emphasis on tumorigenesis. Tumour Biol: J Int Soc Oncodev Biol Med.

[72] Le TX, Munekage Y (2004). Residues of selected antibiotics in water and mud from shrimp ponds in mangrove areas in Viet Nam. Mar Pollut Bull.

[73] Nygaard K, Lunestad B, Hektoen H, Berge J (1992). Resistance to oxytetracycline, oxolinic acid and furazolidone in bacteria from marine sediments. Aquaculture.

[74] Samuelsen O, Lunestad B, Husevag B, Holleland T, Ervik A (1992). Residues of oxolinic acid in wild fauna following medication in fish farms. Dis Aquat Org.

[75] Samuelsen O, Lunestad B, Ervik A (1994). Stability of antibacterial agents in an artificial marine aquaculture sediment studied under laboratory conditions. Aquaculture.

[76] Selvik A, Hansen PK, Ervik A, Samuelsen OB (2002). The stability and persistence of diflubenzuron in marine sediments studied under laboratory conditions and the dispersion to the sediment under a fish farm following medication. Sci Total Environ.

[77] Heuer OE, Kruse H, Grave K, Collignon P, Karunasagar I, Angulo FJ (2009). Human health consequences of use of antimicrobial agents in aquaculture. Clin Infect Dis.

[78] Cabello FC (2006). Heavy use of prophylactic antibiotics in aquaculture: a growing problem for human and animal health and for the environment. Environ Microbiol.

[79] Cole DW, Cole R, Gaydos SJ (2009). Aquaculture: environmental, toxicological, and health issues. Int J Hyg Environ Health.

[80] Love DC, Rodman S, Neff RA, Nachman KE (2011). Veterinary drug residues in seafood inspected by the European Union, United States, Canada, and Japan from 2000 to 2009. Environ Sci Technol.

[81] Stonich SC, Bailey C. Resisting the blue revolution: contending coalitions surrounding industrial shrimp farming. Hum Organ. 2000;59(1):23–36.

[82] Bailey C (1988). The social consequences of tropical shrimp mariculture development. Ocean Shoreline Manag.

[83] Cox M, Johnstone R, Robinson J, Albrecht G (2004). A conceptual model of impacts of environmental change on human well-being. The Airs Waters Places Transdisciplinary Conference on Ecosystem Health in Australia.

[84] Tacon AGJ, Metian M (2009). Fishing for feed or fishing for food: increasing global competition for small pelagic forage fish. Ambio.

[85] Speedy AW (2003). Global production and consumption of animal source foods. J Nutr.

[86.•] Kawarazuka N, Bene C (2011). The potential role of small fish species in improving micronutrient deficiencies in developing countries: building evidence. Public Health Nutr.

[87] Roos N, Wahab MA, Hossain MA, Thilsted SH (2007). Linking human nutrition and fisheries: incorporating micronutrient-dense, small indigenous fish species in carp polyculture production in Bangladesh. Food Nutr Bull.

[88] Weeratunge N, Snyder KA, Sze CP (2010). Gleaner, fisher, trader, processor: understanding gendered employment in fisheries and aquaculture. Fish Fish.

[89] Naylor RL, Goldburg RJ, Primavera JH (2000). Effect of aquaculture on world fish supplies. Nature.

[90] Naylor R, Hindar K, Fleming I (2005). Fugitive salmon: assessing the risks of escaped fish from net-pen aquaculture. Bioscience.

[91] Tacon A, Metian M (2008). Global overview on the use of fish meal and fish oil in industrially compounded aquafeeds: trends and future prospects. Aquaculture.

[92] Naylor R (2005). Aquaculture and ocean resources: raising tigers of the sea. Annu Rev Environ Resour.

[93] Firestone J, Kempton W, Krueger A, Loper C (2004). Regulating offshore wind power and aquaculture: messages from land and sea. Cornell J Law Public Policy.

[94] Naylor R (2006). Offshore aquaculture legislation. Science (New York, NY).

[95] Ayer NW, Tyedmers PH (2009). Assessing alternative aquaculture technologies: life cycle assessment of salmonid culture systems in Canada. J Clean Prod.

[96] Lundin CG. Global attempts to address shrimp disease. The World Bank; 1995.

[97] Bondad-Reantaso M, Subasinghe R (2005). Disease and health management in Asian aquaculture. Vet Parasitol.

[98] Walker PJ, Mohan CV (2009). Viral disease emergence in shrimp aquaculture: origins, impact and the effectiveness of health management strategies. Rev Aquac.

[99] Kibenge FS, Godoy MG, Wang Y (2009). Infectious salmon anaemia virus (ISAV) isolated from the ISA disease outbreaks in Chile diverged from ISAV isolates from Norway around 1996 and was disseminated around 2005, based on surface glycoprotein gene sequences. Virol J.

[100] Mardones FO, Perez AM, Valdes-Donoso P, Carpenter TE (2011). Farm-level reproduction number during an epidemic of infectious salmon anemia virus in southern Chile in 2007–2009. Prev Vet Med.

[101] Murray AG, Peeler EJ (2005). A framework for understanding the potential for emerging diseases in aquaculture. Prev Vet Med.

[102] McClure CA, Hammell KL, Dohoo IR (2005). Risk factors for outbreaks of infectious salmon anemia in farmed Atlantic salmon, *Salmo salar*. Prev Vet Med.

[103] Aslesen HW, Astroza A, Gulbrandsen M. Multinational companies embedded in national innovation systems in developing countries: the case of Norwegian fish farming multinationals in Chile. In: *GLOBELICS 7th International Conference.* Dakar, Senegal; 2009.

[104] De Silva SS, Subasinghe RP, Bueno PB, Phillips MJ, Hough C, McGladdery SE, Arthur JR (2000). A global perspective of aquaculture in the new millennium. Technical Proceedings of the Conference on Aquaculture in the Third Millennium.

[105] Deutsch L, Gräslund S, Folke C (2007). Feeding aquaculture growth through globalization; exploitation of marine ecosystems for fishmeal. Glob Environ Chang.

[106] Wacyk J, Powell M, Rodnic K, Overturf K, Hill R, Hardy R (2012). Dietary protein source significantly alters growth performance, plasma variables and hepatic gene expression in rainbow trout (*Oncorhynchus mykiss*) fed amino acid balanced diets. Aquaculture.

[107] Torstensen BE, Espe M, Stubhaug I, Lie Ø (2011). Dietary plant proteins and vegetable oil blends increase adiposity and plasma lipids in Atlantic salmon (*Salmo salar L.*). Br J Nutr.

[108] Tocher D (2010). Fatty acid requirements in ontogeny of marine and freshwater fish. Aquac Res.

[109] Good J. Replacement of dietary fish oil with vegetable oils: effects on fish health. University of Sterling; 2004.

[110] De Schutter O. The right to food. United Nations; 2012.

[111] Boissy J, Aubin J, Drissi A, van der Werf HMG, Bell GJ, Kaushik SJ (2011). Environmental impacts of plant-based salmonid diets at feed and farm scales. Aquaculture.

[112] Taelman SE, De Meester S, Roef L, Michiels M, Dewulf J (2013). The environmental sustainability of microalgae as feed for aquaculture: a life cycle perspective. Bioresour Technol.

[113] Turchini G, Torstensen B, Ng W (2009). Fish oil replacement in finfish nutrition. Rev Aquac.

[114.•] Watson AM, Barrows FT, Place AR (2013). Taurine supplementation of plant derived protein and n-3 fatty acids are critical for optimal growth and development of cobia, *Rachycentron canadum*. Lipids.

[115] Banerjee A (2011). Food, feed, fuel: transforming the competition for grains. Dev Chang.

[116] EU clears use of some animal proteins in fish feed. In: *The International Magazine of Rendering.* 2013.

[117] Subasinghe R, Soto D, Jia JS (2009). Global aquaculture and its role in sustainable development. Rev Aquac.

[118] Statement on the Aquaculture Stewardship Council (ASC) Standard [http://www.globalsalmoninitiative.org/wp-content/uploads/2013/08/ASC-Statement1.pdf]

[119] Osburn B, Scott C, Gibbs P (2009). One world—one medicine—one health: emerging veterinary challenges and opportunities. Rev Sci Tech.

[120] Wielinga PR, Schlundt J (2013). Food safety: at the center of a One Health approach for combating zoonoses. Curr Top Microbiol Immunol.

[121] Food and Agriculture Organization of the United Nations. FAO global aquaculture production volume and value statistics database updated to 2012. Edited by Fisheries and Aquaculture Department; 2014.

